# Farming, Foreign Holidays, and Vitamin D in Orkney

**DOI:** 10.1371/journal.pone.0155633

**Published:** 2016-05-17

**Authors:** Emily Weiss, Lina Zgaga, Stephanie Read, Sarah Wild, Malcolm G. Dunlop, Harry Campbell, Ruth McQuillan, James F. Wilson

**Affiliations:** 1 Usher Institute for Population Health Sciences and Informatics, University of Edinburgh, Edinburgh, Scotland; 2 Public Health and Primary Care, Trinity College Centre for Health Sciences, Dublin, Ireland; 3 MRC Human Genetics Unit, MRC Institute of Genetics and Molecular Medicine, University of Edinburgh, Edinburgh, Scotland; University of Alabama at Birmingham, UNITED STATES

## Abstract

Orkney, north of mainland Scotland, has the world’s highest prevalence of multiple sclerosis (MS); vitamin D deficiency, a marker of low UV exposure, is also common in Scotland. Strong associations have been identified between vitamin D deficiency and MS, and between UV exposure and MS independent of vitamin D, although causal relationships remain to be confirmed. We aimed to compare plasma 25-hydroxyvitamin D levels in Orkney and mainland Scotland, and establish the determinants of vitamin D status in Orkney. We compared mean vitamin D and prevalence of deficiency in cross-sectional study data from participants in the Orkney Complex Disease Study (ORCADES) and controls in the Scottish Colorectal Cancer Study (SOCCS). We used multivariable regression to identify factors associated with vitamin D levels in Orkney. Mean (standard deviation) vitamin D was significantly higher among ORCADES than SOCCS participants (35.3 (18.0) and 31.7 (21.2), respectively). Prevalence of severe vitamin D deficiency was lower in ORCADES than SOCCS participants (6.6% to 16.2% p = 1.1 x 10^−15^). Older age, farming occupations and foreign holidays were significantly associated with higher vitamin D in Orkney. Although mean vitamin D levels are higher in Orkney than mainland Scotland, this masks variation within the Orkney population which may influence MS risk.

## Introduction

Multiple sclerosis is a chronic, complex disease with genetic, environmental and behavioural factors implicated in its aetiology [1]. Greater distance from the equator is associated with increasing MS prevalence [2]; increasing latitude is also noted for weaker ultraviolet B (UVB) radiation which inhibits cutaneous production of vitamin D [3]. As such, one environmental risk factor is thought to be vitamin D deficiency, however, vitamin D is also a marker for exposure to UV radiation, the benefits of which may be independent of vitamin D production [4–6]. A variety of factors hinder or prevent UVB from reaching the earth's surface, including latitude and weather [7], or from initiating cutaneous vitamin D synthesis, such as sun protection creams and clothing cover.

Although a recent systematic review of vitamin D status worldwide found that vitamin D concentrations do not appear to be dependent upon latitude [8], exposure to ultraviolet B (UVB) radiation from sunshine is the most potent source of vitamin D for humans [9]. In the United States, a latitudinal relationship exists between wintertime vitamin D and wintertime UV doses [10], likely resulting from the few days in which vitamin D can be produced at such latitudes [3, 10]. This latitudinal relationship further reflects the latitudinal MS prevalence distribution in the US [10]. Additionally, a significant relationship between regional UVB radiation and MS prevalence has been noted in France in the French farming population and their families [11]. Increasing MS prevalence was associated with decreasing ambient UVB; the trend was additionally found to be stronger in both wintertime and in women [11]. A similar relationship between decreasing UV and increasing prevalence has been identified in Newfoundland [12] and Australia [13].

Controversy remains regarding the role of vitamin D in chronic conditions; whilst deficiency may have a causal role in the aetiology of some diseases it may also simply be a biomarker for ill health. A body of evidence is however accumulating, suggesting a causal role for vitamin D in MS risk, pathology and progression [14–16]. A recent Mendelian randomisation study found genetically lowered 25-hydroxyvitamin D (25(OH)D) level by one standard deviation in log-transformed 25(OH)D was associated with a two-fold increased risk of MS [16]. Interactions between vitamin D and the major genetic risk factor, HLA-DRB1*1501, have been identified [17], and several rare variants conferring MS risk have been found in the *CYP27B1* gene, which encodes an enzyme which catalyses the conversion of 25(OH)D to the bioactive form [18]. Further, early exposure to vitamin D in-utero and in neonatal mice led to optimal numbers of invariant natural killer T cells [19], deficiency of which are observed in MS patients [19, 20]. Alongside the month-of-birth effect, where children born after a winter gestation are at higher risk of MS [21], the role of adequate in-utero vitamin D increasingly appears to be critical for future autoimmunity.

The beneficial role of UV exposure independent of vitamin D production has been supported in animal studies, using experimental autoimmune encephalomyelitis (EAE) as the model for MS. Continuous treatment with UVB was found to suppress clinical signs of EAE which, although leading to slight elevations in serum 25(OH)D_3_, were insufficient to cause disease suppression by vitamin D [4]. Furthermore, suppression of EAE was found to occur upon irradiation of mice to narrow-band UV light, with a wavelength of between 300 and 315 nm and a peak of effectiveness at 311 nm. As vitamin D requires a wavelength between 270 and 300 nm, optimally between 295 and 300 nm to initiate cutaneous synthesis, the narrow band UV supressing EAE had no effect on 25(OH)D levels [5], strongly suggesting a role of UV exposure independent of vitamin D. In MS, an Australian multi-centre case-control study found higher sun exposure and higher vitamin D levels to be independently associated with lower risk for first demyelinating events [6].

Scotland, between latitudes 54° and 60° north, has inadequate strength of sunshine between October and March for vitamin D synthesis [22]; a cloudy climate year-round further leads to widespread vitamin D deficiency [23] strongly indicating limited UV exposure in the population. The protective effect of supplementation and sunny holidays on 25(OH)D in Aberdeen, a Scottish city at 57° north, has previously been noted in a study of postmenopausal women [24]. Orkney, an isolated archipelago ten to sixty miles from the north coast of Scotland, is an area of exceptionally high MS prevalence [25]. Seventeen of the 70 islands are inhabited with a predominantly rural population totalling 21,349 at the 2011 census. The 2011 census also revealed an ongoing agricultural tradition with 10% of the workforce employed in agriculture or fishing.

**Fig 1:**
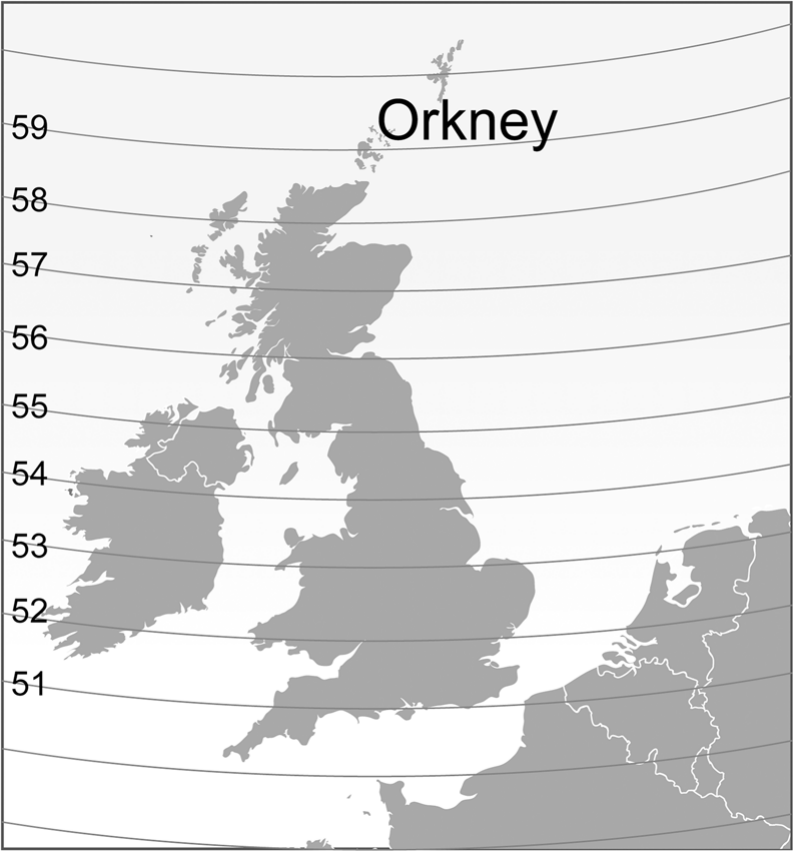
Map of Orkney in relation to the Scottish mainland and north-west periphery of Europe, with 57^th^ and 59^th^ degrees of latitude.

As an independent risk factor, or as a marker of UV exposure, it is important to understand the determinants of plasma vitamin D in the context of MS and other diseases of public health importance. In this study we aimed to describe vitamin D levels in Orkney (Fig 1). This involved identifying the prevalence of vitamin D deficiency in Orkney compared to the Scottish mainland, and establishing the determinants of circulating plasma 25-hydroxyvitamin D (25(OH)D) in Orkney.

## Methods and Materials

### Study Populations

The study population comprised Orkney Complex Disease Study (ORCADES) participants, recruited from 2005 to 2011, and Scottish Colorectal Cancer Study (SOCCS) controls, which ran from 1999 to 2006. Both these studies have been described in detail elsewhere [26, 27]. Briefly, ORCADES, a cross-sectional genetic epidemiology study concerned with identifying genetic factors that influence complex disease, comprised 2078 participants with at least one Orcadian grandparent. SOCCS, a case-control study of colorectal cancer in Scotland, included 2235 adult controls without colorectal cancer, identified from the Community Health Index in Scotland as being aged within 5 years their matched case, of the same sex and living in the same area. All participants provided written, informed consent prior to participation. Both studies have ethical approval from NHS Orkney, NHS Grampian or NHS Lothian.

### 25-hydroxyvitamin D measurement

Fasting blood samples were drawn from ORCADES participants using the Sarstedt Monovette system. Samples were processed and transferred for storage at -40^0^ C, and later -80^0^ C, until analysis. Blood was drawn from all SOCCS participants, processed and transferred for storage at -80^0^ C. Both studies ran over multiple years, and therefore measures per month comprise blood drawn in multiple Januarys, multiple Februarys and so on.

Vitamin D status is determined by measuring circulating 25(OH)D which is generally considered the best indicator of vitamin D status [28]. Both 25(OH)D_2_ and 25(OH)D_3_ were measured using the liquid chromatography-tandem mass spectrometry (LC-MS/MS) method. The total of the two measures was taken for total circulating 25(OH)D, however most samples from both studies contained no 25(OH)D_2_. The lower limit of detection using LC-MS/MS was 10 nmol/L. All samples were measured in the same laboratory following standard protocols; quality control procedures were performed according to current best evidence for 25(OH)D measurement in population studies [29].

A range of cut-offs to define sufficiency and deficiency have been proposed, however in line with other recent studies we considered circulating 25(OH)D of 50 nmol/L or over to be sufficient [30], 25 to 50 nmol/L to be at risk of deficiency or insufficient, and deficiency to be less than 25 nmol/L [31]. We additionally explored those at the lower end of deficiency, where we considered circulating 25(OH)D below 12.5 nmol/L to be severely deficient [32].

### Lifestyle factors

ORCADES participants attended clinics where several biometric measures were recorded. Each participant also completed a medical and lifestyle questionnaire from which vitamin D intake, physical activity (PA) and socioeconomic status (SES) were derived.

BMI was calculated as kg/m^2^ and treated as a continuous variable. Height was taken without shoes and weight wearing only light clothing. Based on questionnaire data we derived a variable encompassing work and leisure time PA throughout the year. Participants classified leisure activity as either 1) light (mostly sitting, light housework) or 2) moderate exercise; and likewise work activity as 1) mostly sitting, 2) mostly standing, 3) manual work or 4) heavy manual work. Dietary vitamin D intake was estimated from two self-administered food frequency questionnaires, the cardiovascular disease questionnaire (CVDQ) and bone density questionnaire (BDQ). The CVDQ was treated as the primary source due to the higher response rate, however information contained in the BDQ that was not present in the CVDQ was merged to create the most comprehensive variable possible. Further, a research nurse-administered drug questionnaire sought information about medications and dietary supplements. Participants also described their frequency of taking holidays within or outside the UK (never, less than once a year, once a year, more than once a year). Although the Scottish Index for Multiple Deprivation (SIMD) is available for Orkney, the scattered and heterogeneous population means that concentrations of poverty or affluence are difficult to identify; moreover neighbouring islands are grouped together in units thus there is little discrimination [33]. Principal Components Analysis (PCA) is a statistical technique to reduce a number of variables into a few independent dimensions reflecting the underlying patterns in the data, and was used here to construct SES indices [34]. To establish a variable that differentiates between individuals, three SES variables were thereby derived from 10 questionnaire items with significant loadings in PCA (S1 Table). Additionally, we applied an occupational prestige score to questionnaire occupation information which was then included in the PCA [35]. Holidays, car age and council tax band loaded significantly onto the first component; housing tenure, length of car ownership and highest qualification loaded significantly onto the second component, and job prestige score, years in education and supervisory role at work loaded significantly onto the third. This third component captures “non-traditional” lifestyles reflecting managerial, administrative and professional positions in contrast to traditional agricultural work. Time outside in summer was summed from participants’ estimates of average time spent without a roof covering on summer work and leisure days. Data from SOCCS included age, sex, month of blood sample and 25(OH)D measurement.

### Statistical analysis

We matched the ORCADES and SOCCS datasets on age to within two years (Table 1) to remove any differences arising from age structures. Matching was carried out blind excepting dataset of origin and age. Because of the large effect of season on vitamin D levels, we standardised 25(OH)D measurements to the month of May; values obtained thereby represent those that would be expected if every sample were drawn in May [32]. The mean of monthly means in the mainland Scottish data is 34.4 nmol/L; in the Orcadian data the mean of monthly means is 37.7 nmol/L. The May means are 33.8 nmol/L and 35.5 nmol/L, respectively. We used May-standardised measurements for all analyses concerned with determinants of vitamin D, and also to compare Orkney and mainland Scotland in deficiency levels. For analyses concerned with vitamin D and time of year we used crude 25(OH)D measures. Data are presented as mean (standard deviation).

**Table 1:** Distribution of age and crude vitamin D in age-matched Orkney and mainland Scotland datasets. The mainland dataset excludes people from above the 57^th^ degree of latitude.

	Orkney	Mean 25OHD	Mainland	Mean 25OHD
	No (%)	(nmol/L)	No (%)	(nmol/L)
Participants	1453	36.2	1453	35.4
< 40	46 (3.17)	26.8	46 (3.17)	36.5
40–49	263 (18.1)	33.4	263 (18.1)	39.5
50–59	399 (27.5)	35.7	400 (27.5)	38.7
60–69	466 (32.1)	37.9	464 (31.9)	33.3
70 +	279 (19.2)	39.5	280 (19.3)	30.4
Sex (male)	590 (40.6)	37.2	794 (54.6)	35.4
Sex (female)	863 (59.4)	35.6	659 (45.4)	35.4

We plotted crude 25(OH)D by location as a density plot and by month, and compared using a t-test. We compared vitamin D by age group using t-tests. To compare levels of deficiency in Orkney and mainland Scotland, we divided participants into groups of deficiency and plotted May-adjusted vitamin D for each deficiency group and location and tested for differences using chi-square tests.

For determinants of May-adjusted 25(OH)D in Orkney, we ran a series of bivariable models of May-adjusted 25(OH)D against environmental and demographic variables of interest. Those that were significant were put into a multivariable model. These significant variables comprised BMI, age at venepuncture, foreign holidays, PA, SES, dietary vitamin D and working status. Sex was also a covariate. A large percentage of missing data (S2 Table) was imputed using Multiple Imputation of Chained Equations (MICE)[36] after excluding 28 individuals with missing outcome data. We ran 68 cycles of 100 imputations and pooled the results in a linear regression model. We ran the same model using complete cases only. Statistical tests were two-sided with p<0.05 taken as significant. Finally, we applied a one-way ANOVA to compare mean May-adjusted 25(OH)D across the three groups of participants that we identified as a result of our analyses.

We assessed homoscedasticity by inspection of a QQ plot, and distribution of residuals using a histogram with superimposed normal curve. Independence was checked using the Durbin Watson statistic, multicollinearity and outliers using the vif statistic and Cook’s distance, respectively. All analyses were conducted using R software version 3.2.0 [37].

## Results

For this study, 64 individuals were excluded from ORCADES who were not resident in Orkney, 10 who had MS, as well as 8 duplicate measures. Characteristics of ORCADES participants are presented in Table 2. Twenty-three people were excluded from the Scottish Colorectal Cancer Study who lived above the 57^th^ degree of latitude. For comparison analyses, data were age-matched giving a final count of 1453 people in each dataset.

**Table 2:** Characteristics of ORCADES Study participants (n = 1972).

Characteristic	No or mean	SD	% or range
Age at venepuncture (years)	53.4	15.3	16.5–100.2
Sex			
Female	1191	-	60.4
Male	781	-	39.6
Body Mass Index (kg/m^2^)	27.7	4.9	16.9–53.9
Vitamin D intake (μg)	4.4	3.1	0.00–34.1
Physical activity*	5.1	1.2	3.0–8.0
Summer minutes outdoors	223	142	4.8–900
Working	1367	-	69.3
Retired	547	-	27.7
Holidays outside the UK			
< once a year	1472	-	74.6
Once a year	329	-	16.7
> once a year	105	-	5.3
Years in education	16	1.2	1.0–23
Qualification level			
O & standard grades, CSE	275	-	13.9
Highers, A levels	787	-	39.9
Certificates/diplomas	739	-	37.5
Bachelor/Master degree	88	-	4.5
Doctorate	13	-	0.7

* Physical activity scored from 1 (mostly sitting; inactive) to 4 (heavy manual labour; active) across different domains within work and leisure. Each score is the sum of answers creating an individual value for each participant.

**Fig 2:**
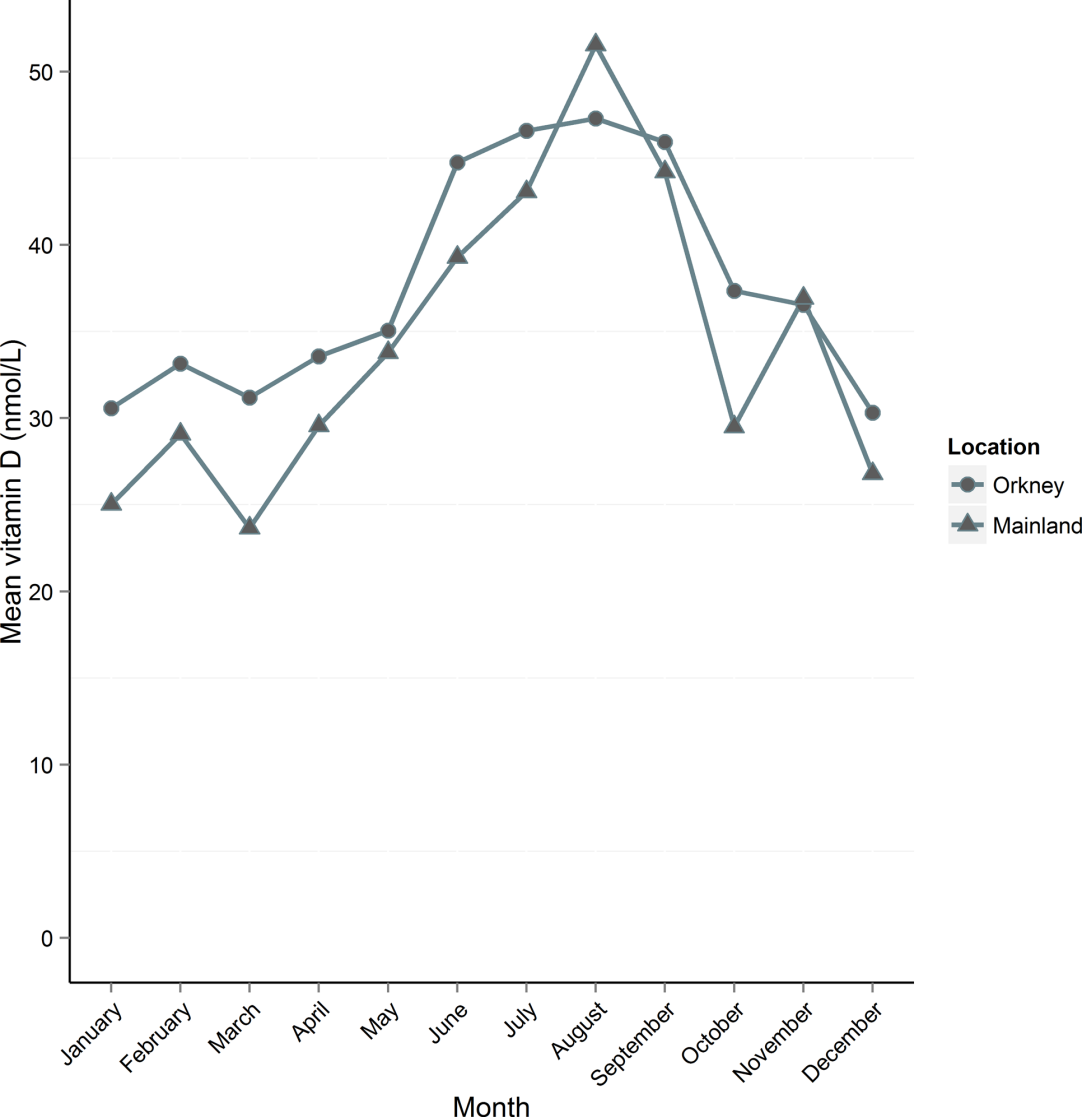
Mean crude vitamin D concentration (nmol/L) per month by location, using age-matched data. Orkney’s mean vitamin D is higher than the mainland for every month except August and November. Each study ran over consecutive years and measurements taken in the same month each year were pooled.

**Fig 3:**
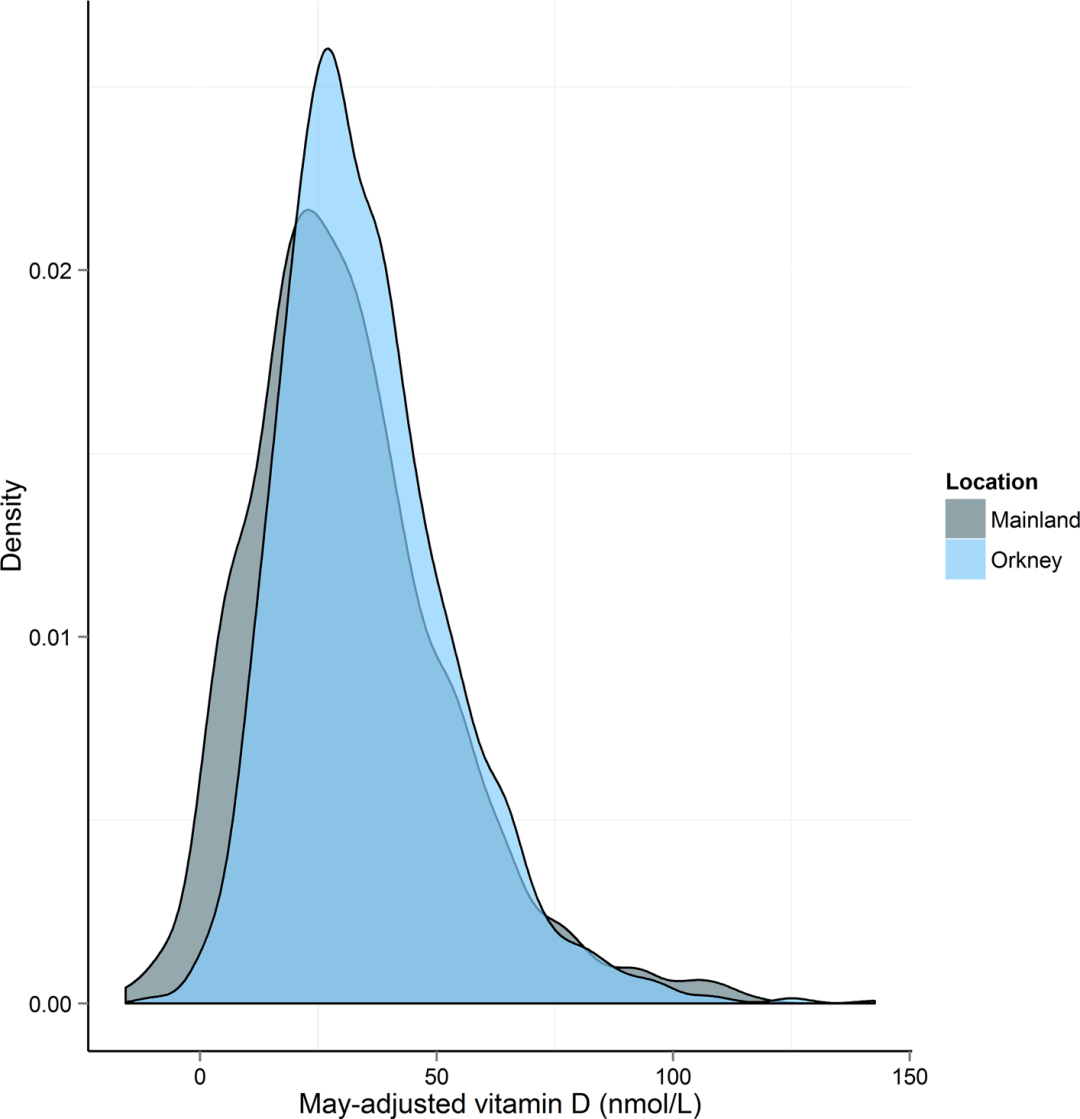
Comparison of May-adjusted vitamin D distribution in Orkney and mainland Scotland using age-matched data. The distribution for Orkney is to the right of the distribution for the mainland, reflecting the lower prevalence of severe deficiency, and peaks higher.

**Fig 4:**
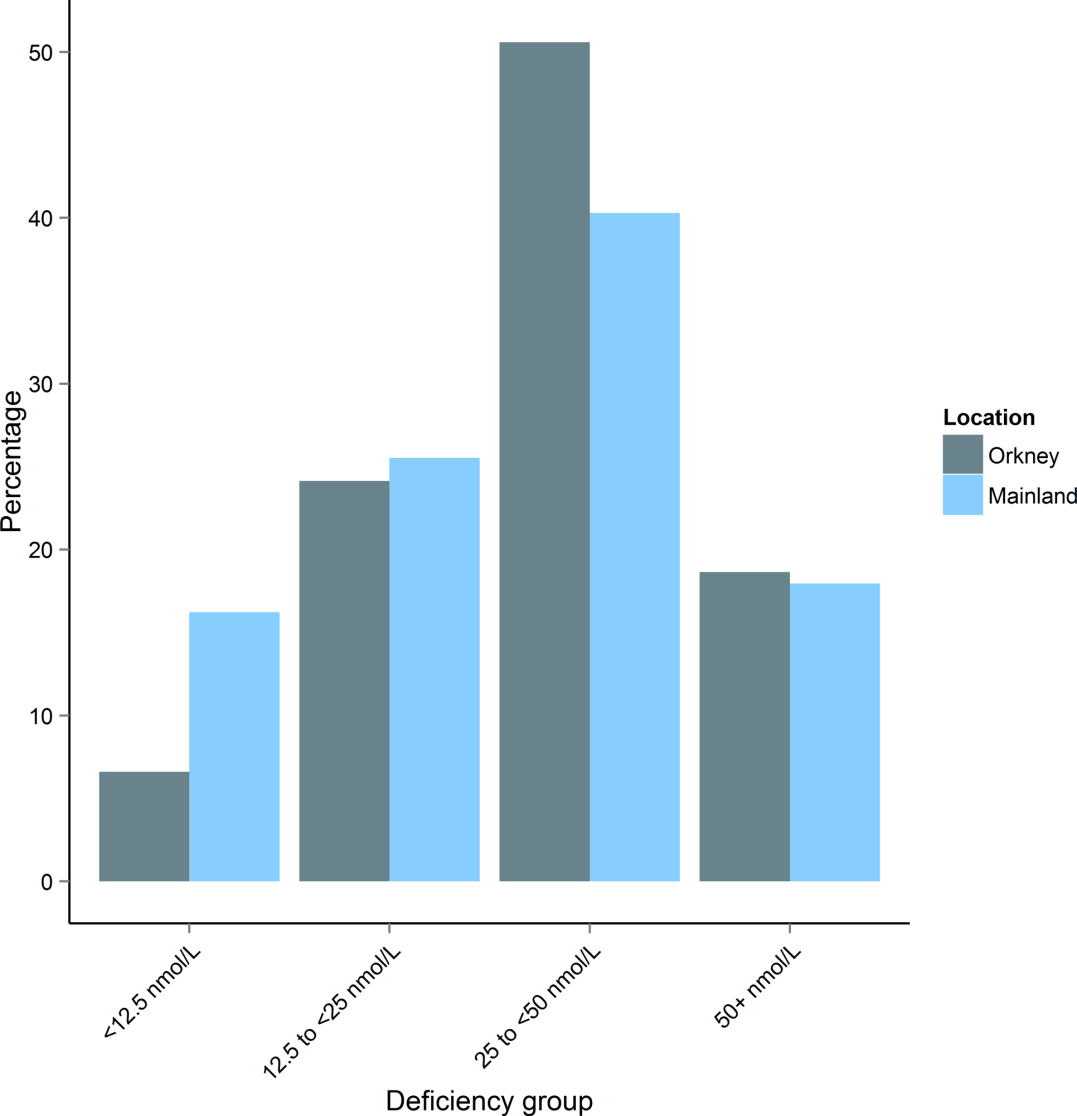
Comparison of percentage of people in May-adjusted vitamin D deficiency groups by location. The main differences occur in the severely deficient group (<12.5 nmol/L) which has significantly fewer people from the Orkney sample (χ^2^(1) = 64.2, *p* = 1.10 x 10^−15^), and the ‘at risk’ category (25-<50 nmol/L) which has significantly fewer people from the mainland Scottish sample (χ^2^(1) = 30.3, *p* = 3.78 x 10^−8^).

Using age-matched data we compared mean 25(OH)D in Orkney to mainland Scotland (Fig 2). Orkney had significantly higher crude 25(OH)D than mainland Scotland (Orkney 35.3 (18.01), Mainland 31.7 (21.18), *t*(2800) = -4. 93, p = 8.5 x 10^−7^). Mean 25(OH)D was higher in Orkney for every month except August when the mainland peaks at ~50 nmol/L. The distribution of vitamin D levels is shifted to the right in Orkney (Fig 3). We compared vitamin D in Orkney and mainland Scotland by age group (Table 3). Results for each age group are significantly different, however in the under 40s, 40 to 49 and 50 to 59 age groups, mainland Scotland has higher vitamin D, whilst in the 60 to 69 and over 70s age groups, Orkney has significantly higher vitamin D. Comparing Orkney with the mainland by deficiency group, we found that more people in Orkney had insufficient vitamin D, χ^2^ (1, N = 2863) = 30.3, p = 3.8 x 10^−8^; however, Orkney had significantly fewer people with circulating 25(OH)D of <12.5nmol/L (severely deficient) compared with the mainland, χ^2^ (1, N = 2863) = 64.3, p = 1.1 x 10^−15^ (Fig 4).

**Table 3:** Comparison of vitamin D in Orkney and mainland Scotland by age group.

	N = Orkney; Scotland	Orkney mean crude 25(OH)D	Mainland mean crude 25(OH)D	t-test	p-value
< 40	46; 46	26.8	36.5	-2.67	0.009
40–49	263; 263	33.4	39.5	-3.13	0.002
50–59	399; 400	35.7	38.7	-1.97	0.049
60–69	466; 464	37.95	33.28	3.59	0.0004
70+	279; 280	38.51	30.39	4.84	1.6x10^-6^

To explore correlates of vitamin D in Orkney we ran two multivariable regression analyses, using both imputed data and complete cases. Each model yielded similar results (Table 4). Variables significantly associated with higher 25(OH)D included lower BMI, more foreign holidays, older age and increased PA. Associated with lower 25(OH)D was the “non-traditional” SES grouping.

**Table 4:** Results of linear regression for complete cases and imputed data using May-adjusted vitamin D as the outcome.

	Multivariable models			
	Model 1^a^ (n = 628)		Model 2^b^ (n = 1949)	
Predictors	Est (95% CI)	p-value	Est (95% CI)	p-value
Intercept	32.4 (18.5, 46.2)	5.25x10^-6^	30.3 (22.2, 38.3)	2.2x10^-13^
Body mass index (kg/m^2^)	-0.75 (-1.02, -0.47)	1.95x10^-7^	-0.54 (-0.70, -0.38)	7.5x10^-11^
Holidays outside the UK				
< once a year	-0.93 (-4.38, 2.52)	0.59	0.75 (-1.34, 2.85)	0.48
Once year	5.03 (0.20, 9.86)	0.041	6.47 (3.47, 9.47)	0.000024
> once a year	18.7 (11.3, 26.2)	1.04x10^-6^	13.5 (9.07, 18.0)	3.4x10^-9^
Age at venepuncture	0.24 (0.11, 0.36)	0.0003	0.14 (0.07, 0.22)	0.00030
Physical activity	1.66 (0.58, 2.75)	0.003	1.42 (0.65, 2.19)	0.00032
Socio-economic status 3 (“non-traditional”)	-2.10 (-3.57, -0.64)	0.005	-1.74 (-2.71, -0.78)	0.00043
Summer minutes outside	0.0046 (-0.00398, 0.013)	0.29	0.006 (-0.00028, 0.012)	0.062
Socio-economic status 2	0.32 (-1.30, 1.94)	0.70	0.69 (-0.34, 1.72)	0.19
Vitamin D intake (μg)	0.201 (-0.18, 0.60)	0.30	0.14 (-0.17, 0.46)	0.37
Working (not retired)	-2.07 (-6.61, 2.47)	0.37	-0.18 (-2.45, 2.09)	0.88
Sex (male)	1.06 (-1.55, 3.67)	0.43	-0.12 (-1.77, 1.52)	0.88
Socio-economic status 1	-0.013 (-1.85, 1.82)	0.99	-0.075 (-1.17, 1.02)	0.89

^a^ Model 1 constructed using complete cases in the original dataset, R^2^ = 0.204.

^b^ Model 2 constructed using 68 datasets with missing data completed by imputation (100 cycles), R^2^ = 0.111 (People missing outcome data were excluded from the imputation model).

Socio-economic status was derived from principal components analysis.

**Fig 5:**
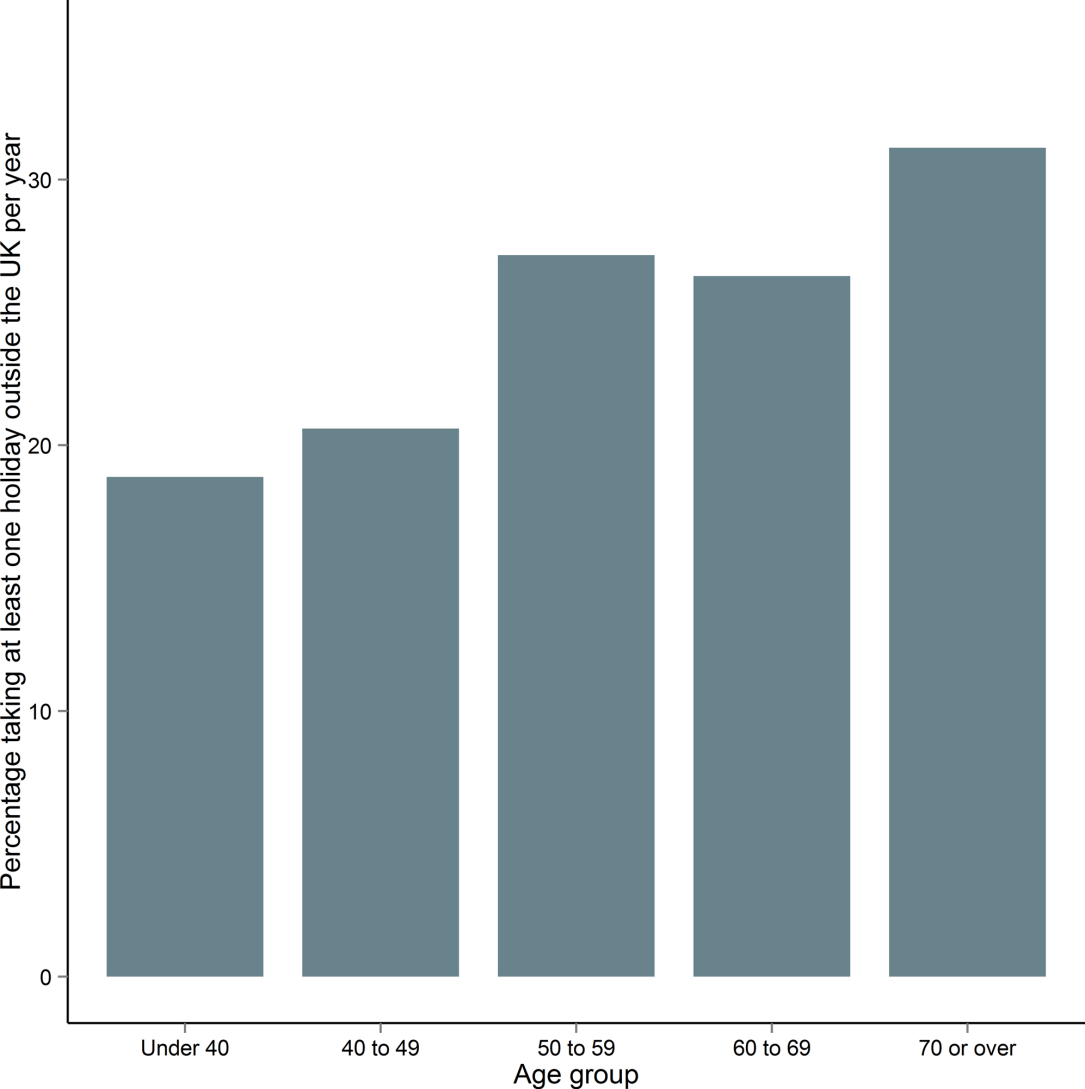
Percentage of people per age group in ORCADES who holiday outside the UK at least once a year. People over 50 take significantly more holidays than those under 50.

The association between older age and higher vitamin D required further exploration; we began by comparing foreign holiday-takers and their non-holidaying counterparts. We found that people over 50 were significantly more likely to take foreign holidays at least once a year compared to people under 50 (Table 5, Fig 5) (χ^2^(1) = 6.4, *p* = 0.0083). We termed this the ‘Saga’ effect. Additionally, we found that foreign holidays had a stronger effect on people over 50 who had their blood drawn in the low vitamin D (weaker UVB) season (October to March) compared with people who had their blood drawn in the high vitamin D (stronger UVB) season (April to September) (Table 6).

**Table 5:** Comparison of people over 50 who holiday outside the UK at least once a year (n = 281) and people over 50 who holiday outside the UK less than once a year or never (n = 851). Unpaired t-tests applied to continuous data; chi-square tests applied to categorical data.

	Over-50s, holiday	Over-50s, no holiday	t-test or	p-value
	No (%) or Mean (SD)	No (%) or Mean (SD)	Chi-square	
Socio-economic status 1	1.01 (0.76)	-0.30 (0.92)	-22.6	<2.2x10^-16^
Job prestige score	0.32 (1.08)	-0.19 (0.96)	-7.09	5.1x10^-12^
Socio-economic status 3 (“non-traditional”)	0.18 (1.05)	-0.29 (0.92)	-6.50	2.1x10^-10^
Supervisory role at work				
Yes	186 (66.4)	377 (45.1)		
No	94 (33.6)	459 (54.9)	38.2	6.4x10^-10^
Years in education	15.9 (1.26)	15.5 (1.14)	-5.39	1.1x10^-6^
Body mass index (kg/m^2^)	27.6 (3.91)	28.9 (5.03)	4.41	1.2x10^-5^
Highest qualification				
O & standard grades, CSE*	33 (11.8)	102 (12.1)		
Highers, A levels**	114 (40.7)	246 (29.3)		
Certificates/diplomas	113 (40.4)	461 (54.8)		
Bachelor/Master/PhD	20 (7.14)	32 (3.81)	22.2	5.8x10^-5^
Summer minutes	252 (146)	214 (136)	-3.38	0.001
Age	62.9 (7.62)	64.4 (8.63)	2.55	0.01
Bodyfat %	33.7 (7.51)	34.9 (8.07)	2.34	0.02
Socio-economic status 2	0.43 (0.76)	0.32 (0.76)	-2.06	0.04
Vitamin D intake (μg)	5.09 (3.60)	4.78 (3.13)	-1.12	0.26
Physical activity	5.21 (1.20)	5.16 (1.26)	-0.37	0.71

* School examinations taken in the UK ~16 years of age

** School examinations taken in the UK ~18 years of age

**Table 6:** Mean May-adjusted vitamin D (nmol/L) according to season of venepuncture (high season (April-September, n = 96) vs low season (October-March, n = 185)) in people over 50 who take a holiday outside the UK at least once a year. Linear regression with May-adjusted vitamin D as the outcome.

	Std Beta	Beta (95% CI)	Std error	p-value
High season over-50s	0.17	7.86 (3.57–12.2)	2.18	0.00035
Low season over-50s	0.19	8.33 (5.23–11.4)	1.57	1.75x10^-7^

To explore under-40s further, we ran the same analyses comparing those who do and do not holiday outside the UK that were used for over-50s (S3 Table). The same results were significant, excepting body fat percentage, socio-economic status 2, age, and summer minutes spent outside.

We also identified a ‘farmer effect’ (Table 7). Participants employed in “traditional” agricultural occupations that kept them outdoors had significantly higher mean vitamin D levels than participants in non-traditional professions that kept them indoors (farmers 36.9 (18.0), non-farmers 33.8 (11.8), *t*(383) = 2.46, p = 0.014). Further, farmers tended to be older.

**Table 7:** Comparison of farmers (n = 265) and non-farmers (n = 1649) on variables of interest in Orkney. Farmers are anyone who identified their primary profession as farmer. Unpaired t-tests applied to continuous data; chi-square tests applied to categorical data.

	Farmers	Non-farmers	t-test or	p-value
	No (%) or Mean (SD)	No (%) or Mean (SD)	Chi-square	
Age	60.7 (11.4)	52.4 (11.5)	-8.86	<2.2x10^-16^
Socio-economic status 3 (“non-traditional”)	-0.30 (0.58)	0.10 (1.02)	8.77	<2.2x10^-16^
Years in education	15.4 (1.01)	16.1 (1.24)	10.16	<2.2x10^-16^
Socio-economic status 1	-0.44 (1.00)	0.09 (0.97)	7.86	5.4x10^-14^
Physical activity	5.87 (1.23)	5.10 (1.21)	-7.60	8.9x10^-13^
Highest qualification				
O & standard grades, CSE*	24 (9.2)	251 (15.3)		
Highers, A levels**	84 (32.1)	702 (42.8)		
Certificates/diplomas	153 (58.4)	586 (35.8)		
Bachelor/Master/PhD	1 (0.4)	100 (6.1)	55.9	4.3x10^-12^
Bodyfat %	30.4 (8.7)	33.1 (8.6)	4.51	9.1x10^-6^
Supervisory role at work				
Yes	109 (41.3)	866 (53.2)		
No	155 (58.7)	763 (46.8)	12.8	0.0003
Socio-economic status 2	0.28 (0.92)	0.07 (0.92)	-3.39	0.0008
Body mass index (kg/m^2^)	28.4 (4.80)	27.7 (4.96)	-2.12	0.04
Vitamin D intake (μg)	4.71 (2.93)	4.40 (3.20)	-1.29	0.19
Summer minutes	236 (151)	221 (141)	-1.21	0.23

* School examinations taken in the UK at ~16 years of age

** School examinations taken in the UK at ~18 years of age

**Fig 6:**
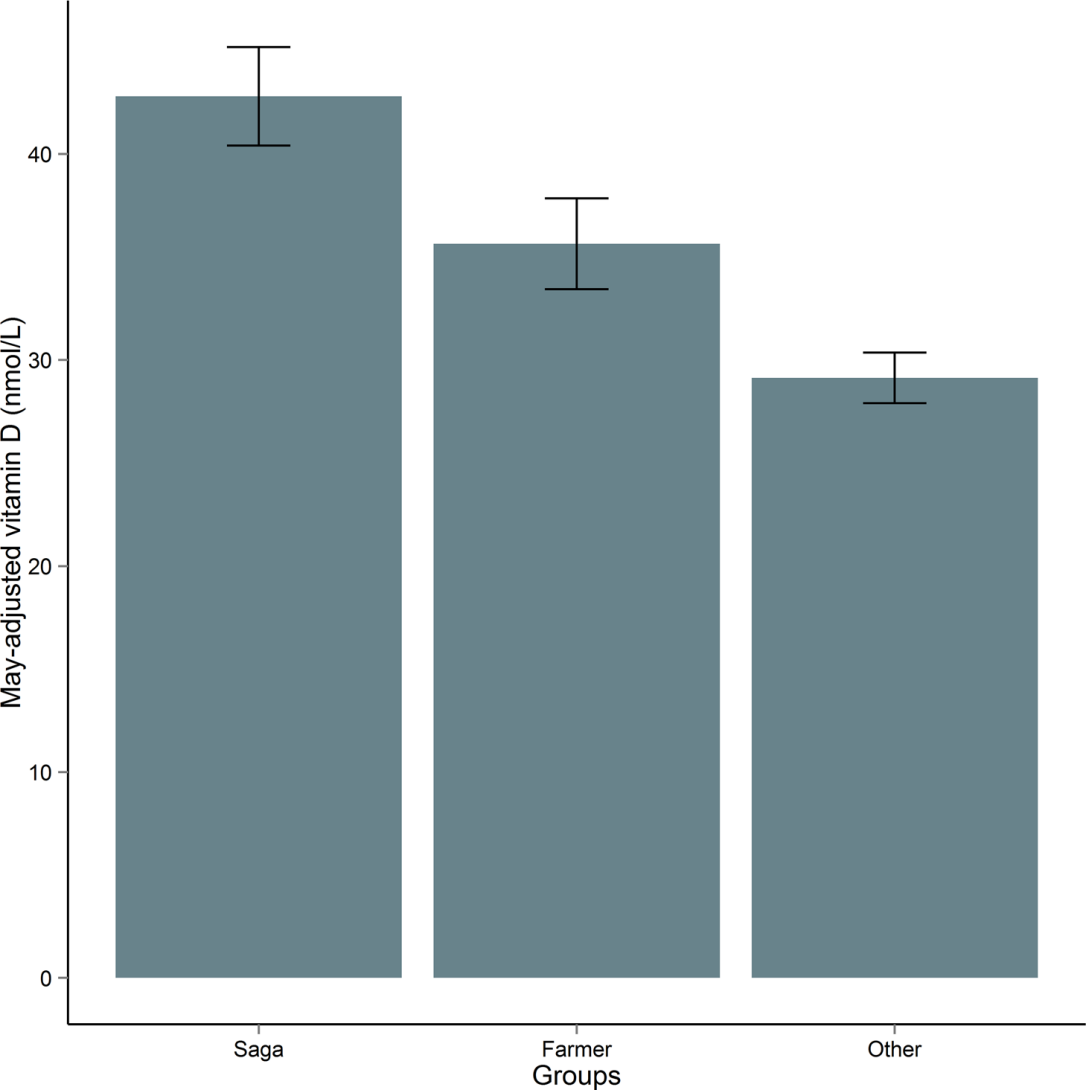
Mean May-adjusted vitamin D (nmol/L) in different groups in ORCADES. 95% confidence interval bars are given. “Saga” refers to people over 50 who take a holiday outside the UK at least once a year; “Farmer” to people who identified their primary profession as farming; “Other” is people under 50 who take a holiday outside the UK less than once a year, and are not farmers.

To test for differences in mean vitamin D across the ‘Saga’ group, farmers, and non-farmers who are under 50 and do not take foreign holidays, we did a one-way ANOVA. This ANOVA was significant: Welch's F(2, 481.99) = 54.49, p = 2.2 x 10^−16^, and we therefore concluded that vitamin D varies significantly across these groups with people over 50 who take foreign holidays having higher vitamin D than farmers, who had higher vitamin D than non-farmers and people under 50 who remain in the UK (Fig 6).

## Discussion

We aimed to compare vitamin D levels in Orkney and mainland Scotland, and to identify the determinants of Orkney vitamin D. Definitions of vitamin D deficiency are much discussed, however it has been proposed that circulating 25(OH)D above 50 nmol/L are sufficient [30, 38]. Vitamin D status in Scotland has been previously explored [32]. Deficient and high risk individuals comprised 63.4% in the previous study; in our Orkney dataset deficient and high risk individuals comprised 65.3%. However, people with severe deficiency (<12.5 nmol/L) comprised 11.8% of the former study and only 5.0% of the latter. Therefore although perhaps initially surprising that mean vitamin D was higher in Orkney despite the higher latitude, the smaller percentage of people with severe deficiency in Orkney led to this elevation. In both datasets the majority are either deficient or at risk of deficiency which could have significant health implications [9].

Ability to synthesise vitamin D decreases with age [39]; it is well established that older age is associated with lower vitamin D and increased deficiency risk [32, 40]. However, we found that lifestyle factors particular to Orkney contributed to better vitamin D in older compared to younger people.

Participants who took foreign holidays had higher vitamin D than those who did not take foreign holidays; furthermore, taking foreign holidays increased by age group. Less than 20% of under-40s in our Orkney sample took foreign holidays, whereas over 30% in the 70 and over group reported leaving the UK at least once a year. Weak sunshine within the UK leads to fewer opportunities for UV exposure and UVB-mediated vitamin D synthesis, and the effect of foreign holiday sun exposure has been previously associated with improved vitamin D in Scotland [24]. Orkney mean vitamin D remained higher than mainland Scotland throughout the year with the exception of two months: August and November. Although we were unable to explore this further, mainland Scotland’s August elevation of vitamin D could be attributed to the effect of holidays, following one month after school holidays. In November, however, mainland Scotland’s mean vitamin D levels were only minimally higher than Orkney. We found that mean vitamin D in people 50 and over taking foreign holidays was significantly higher than vitamin D levels in the rest of the sample. Foreign holidays contributed more to vitamin D levels on blood drawn in months of weaker UVB. We were unable to explore at what time of year people take holidays, however this finding suggests that foreign holidays become more important as a source of UV exposure and therefore vitamin D for Orkney residents in winter. As older people tend to have more freedom to travel outside peak season, they are able to derive most benefit by seeking sun in seasons of scarce sunshine in Orkney. People over 50 who take foreign holidays were found to differ from those who do not take holidays mainly in variables denoting financial security. They were also more likely to have lower BMI and body fat percentage suggesting possible healthier lifestyles than their non-holidaying counterparts.

We also examined under 40s, the age group in which MS is most likely to be diagnosed and pregnancies are most likely to occur, thereby potentially conferring risk to the unborn child. In this group, we found that the main differences in those who do or do not take holidays out of the UK are related to financial security. Only 75 of the 400 people who are under 40 reported leaving the UK for a holiday at least once a year, therefore, in the most at-risk group, inadequate UV exposure in Orkney is compounded by a low prevalence of foreign holidays.

The ‘non-traditional’ SES group derived from PCA, comprising job prestige score, education years and supervisory role at work, was associated with vitamin D. These variables, reflecting “non-traditional” lifestyles of managerial, administrative and professional occupations in contrast to traditional agricultural work, related to farmers and non-farmers. Farmers were found to be slightly less educated, possibly as a result of leaving school at the minimum leaving age about half a year to a year before the first set of examinations. Farmers were also less likely to describe themselves as having a supervisory role at work than non-farmers, and also had a slightly lower-than-average job prestige score. The inverse association between vitamin D and a higher score in this variable means that farmers, who scored lower, had better vitamin D. Farmers in our cohort were also more likely to be older than non-farmers, further contributing to our finding of vitamin D increasing with age.

Physical inactivity and obesity have previously been related to low vitamin D in a large American cohort [41]; the association between lower BMI and higher 25(OH)D is also well established [42]. The mechanism for lower vitamin D in the presence of higher BMI is thought to be a result of increased deposition of vitamin D in body fat [43], making BMI a proxy for adiposity. However, BMI does not distinguish between body fat and fat free mass, and is not always a reliable indicator of adiposity in people with lower body fat but greater muscle mass [44]. The farmers in our cohort reflected this difficulty: they had lower body fat percentage but higher BMI than non-farmers. We found farmers were more active and leaner than non-farmers and it is perhaps therefore fair to assume they have higher than average muscle mass. Nevertheless, in the multivariable models BMI followed what is expected.

Farmers had mean vitamin D significantly higher than non-farmers, but significantly lower than people over 50 who take foreign holidays. Summer minutes outside was not significant in the multivariable analyses, however we performed a t-test for farmers versus non farmers and time spent outside. Farmers, perhaps unsurprisingly, were found to spend significantly more time outside than non-farmers which enables maximisation of even the smallest window of vitamin-D strength sunshine. Although Zgaga et al. found higher vitamin D consumption led to slight improvements in plasma vitamin D [32], we found that diet was not associated with vitamin D in Orkney. However, difficulties involved in building a variable with the available data likely contributed to this finding.

Both studies were recruited on an ‘opt in’ basis which may result in the samples representing a healthier than average population; however a strength of this study was the large number of participants in each cohort. Particularly novel was the number of farmers in our Orkney cohort, enabling us to explore vitamin D in a select group within a rural population which is not often studied. All vitamin D samples from both cohorts were analysed in the same laboratory using the same procedures, helping maintain consistency and reliability of results. We had access to a variety of detailed measures to explore vitamin D in Orkney; however data on time spent outside and vitamin D intake were somewhat limited and these may thus be more strongly implicated in vitamin D than we were able to detect. Nevertheless, we found significant effects and reliable relationships for vitamin D with foreign holidays, BMI, physical activity and age.

## Conclusion

Mean vitamin D in Orkney was higher than mainland Scotland, driven largely by a lower percentage of individuals with severe deficiency in Orkney. Overall concentrations in both cohorts were low with most people either deficient or at risk of deficiency, suggesting that UV exposure for much of the year is low. Older Orkney residents were more likely to have better vitamin D than younger residents, largely resulting from the ‘Saga’ and ‘Farmer’ effects. Those most at risk of deficiency in Orkney were under 40, an age group traditionally considered at lower risk of deficiency, but at increased risk of MS diagnosis. Within these main child-bearing years, a lack of UV exposure and vitamin D deficiency may result in significant autoimmune implications for offspring. The significant contribution of foreign holidays to Orkney vitamin D is consistent with the findings of previous UK studies; the importance of foreign holidays in providing adequate UV exposure to UK residents is underappreciated. We have found that younger ages are more at risk from inadequate UV exposure and vitamin D deficiency in Orkney, a county with a very high prevalence of MS. Further research exploring the relationship between vitamin D and quantitatively-measured exposure to UV radiation from sunshine and physical activity, as well as more detailed dietary information in Shetland, the most northerly UK county with an MS prevalence lower than Orkney, would help further elucidate the roles of UV exposure and vitamin D as MS risk factors in these islands.

## Supporting Information

S1 Table Principal components for socio-economic status variables. The most significant loadings are in bold.
(DOCX)

S2 Table Missing data in the Orkney dataset in variables of interest (DOCX)

S3 Table Comparison of people under 40 who holiday outside the UK at least once a year (n = 75) and people under 40 who holiday outside the UK less than once a year or never (n = 325). Unpaired t-tests applied to continuous data; chi-square tests applied to categorical data.
(DOCX)
