# Supplementary material for: Farming, Foreign Holidays, and Vitamin D in Orkney
Source: PLoS One. 2016 May 17;11(5):e0155633. doi: 10.1371/journal.pone.0155633 (PMC4871509; doi:10.1371/journal.pone.0155633)
Supplement: S1 Table — The most significant loadings are in bold. (DOCX) [file pone.0155633.s001.docx]

**Supplementary table 1.** Principal components for socio-economic status variables. The most significant loadings are in bold.

|  | PC1 | PC3 | PC2 |
| --- | --- | --- | --- |
| Holidays in the UK | **0.70** | 0.17 | 0.01 |
| Holidays outside the UK | **0.65** | 0.28 | 0.16 |
| Car age | **-0.55** | 0.14 | -0.45 |
| Council tax band | **0.45** | 0.24 | -0.25 |
| Boat ownership | 0.33 | 0.05 | -0.02 |
| Job prestige score | 0.16 | **0.76** | 0.21 |
| Years in education | 0.16 | **0.72** | -0.19 |
| Supervisory role at work | 0.16 | **0.52** | 0.16 |
| Housing tenure | 0.08 | 0.07 | **0.67** |
| Length of car ownership | 0.01 | 0.03 | **0.59** |
| Highest qualification | -0.28 | 0.27 | **0.41** |
| Eigenvalue | 2.34 | 1.27 | 1.06 |
| % Explained | 15.3 | 14.7 | 12.4 |
| % Cumulative | 15.3 | 30.0 | 42.6 |
