# Supplementary material for: Farming, Foreign Holidays, and Vitamin D in Orkney
Source: PLoS One. 2016 May 17;11(5):e0155633. doi: 10.1371/journal.pone.0155633 (PMC4871509; doi:10.1371/journal.pone.0155633)
Supplement: S2 Table — (DOCX) [file pone.0155633.s002.docx]

**Supplementary table 2.** Missing data in the Orkney dataset in variables of interest

| Variable | N missing |
| --- | --- |
| May-adjusted vitamin D* | 23 |
| Age at venepuncture | 0 |
| Sex | 0 |
| Body mass index (kg/m^2^) | 72 |
| Physical activity | 669 |
| Vitamin D intake | 703 |
| Summer minutes outside | 531 |
| Work/retired | 58 |
| Holidays outside the UK | 66 |
| SES1 | 268 |
| SES2 | 268 |
| SES3 (“non-traditional”) | 268 |

Note: People with missing outcome data excluded from multiple imputation model
