# Supplementary material for: Farming, Foreign Holidays, and Vitamin D in Orkney
Source: PLoS One. 2016 May 17;11(5):e0155633. doi: 10.1371/journal.pone.0155633 (PMC4871509; doi:10.1371/journal.pone.0155633)
Supplement: S3 Table — Unpaired t-tests applied to continuous data; chi-square tests applied to categorical data. (DOCX) [file pone.0155633.s003.docx]

|  | Under 40s holiday  No (%) or Mean (SD) | Under 40s, no holiday  No (%) or Mean (SD) | t-test or Chi-square | p-value |
| --- | --- | --- | --- | --- |
| Socio-economic status 1 | 0.70 (0.0.75) | -0.258 (0.0.804) | -9.15 | <6.4x10^-15^ |
| Highest qualification |  |  |  |  |
| O & standard grades, CSE | 7 (9.33) | 62 (19.2) |  |  |
| Highers, A levels | 31 (41.3) | 183 (56.7) |  |  |
| Certificates/diplomas | 26 (34.7) | 65 (20.1) |  |  |
| Bachelor/Master/Phd | 11 (14.7) | 13 (4.03) | 23.1 | 3.7x10^-05^ |
| Socio-economic status 3 (“non-traditional”) | 0.94 (1.04) | 0.31 (0.9) | -4.44 | 2.6x10^-05^ |
| Supervisory role at work |  |  |  |  |
| Yes | 51 (69.9) | 149 (46.6) |  |  |
| No | 22 (30.1) | 171 (53.4) | 12.91 | 0.0003 |
| Years in education | 17.2 (1.2) | 16.6 (1.3) | -3.56 | 0.0005 |
| Job prestige score | 0.48 (1.1) | -0.002 (0.9) | -2.98 | 0.004 |
| Body mass index (kg/m^2^) | 24.8 (3.8) | 26.0 (4.9) | 2.37 | 0.019 |
| Bodyfat % | 27.1 (7.5) | 28.5 (9.1) | 1.36 | 0.18 |
| Physical activity | 5.01 (1.2) | 5.18 (1.27) | 1.03 | 0.31 |
| Socio-economic status 2 | -0.38 (1.06) | -0.51 (1.04) | -0.902 | 0.37 |
| Vitamin D intake (μg) | 3.60 (2.6) | 3.45 (3.2) | -0.32 | 0.75 |
| Age | 31.9 (6.3) | 32.04 (6.3) | 0.168 | 0.87 |
| Summer minutes | 219 (148) | 220 (138) | 0.034 | 0.97 |

**Supplementary table 3.** Comparison of people under 40 who holiday outside the UK at least once a year (n=75) and people under 40 who holiday outside the UK less than once a year or never (n=325). Unpaired t-tests applied to continuous data; chi-square tests applied to categorical data.
